# Validación de instrumento para medir el nivel de conocimiento de estudiantes de posgrado y docentes universitarios sobre la ley de trabajo del cirujano dentista

**DOI:** 10.21142/2523-2754-1003-2022-126

**Published:** 2022-09-28

**Authors:** Mauricio A Zapata Sifuentes, Martín A Chávez Méndez

**Affiliations:** 1 Carrera de Estomatología, Universidad Científica del Sur. Lima, Perú. mauriciozapsif96@gmail.com, mchavezme@cientifica.edu.pe Universidad Científica del Sur Carrera de Estomatología Universidad Científica del Sur Lima Peru mauriciozapsif96@gmail.com mchavezme@cientifica.edu.pe

**Keywords:** conocimiento, trabajo, ley, knowledge, work, law

## Abstract

**Objetivo:**

Construir y validar un instrumento para medir el nivel de conocimiento de los estudiantes de posgrado y docentes de la Carrera de Estomatología de la Universidad Científica del Sur sobre la Ley que regula el trabajo del cirujano dentista en el territorio peruano (Ley N.° 27878).

**Materiales y métodos:**

La muestra fue de 38 cirujanos dentistas, participantes del programa de posgrado (20) y profesores (18) de la Carrera de Estomatología de la Universidad Científica del Sur. Se elaboró un cuestionario estructurado de 15 preguntas con base en los artículos de la Ley N.° 27878.

**Resultados:**

Fue aprobado por juicio de expertos y sometido a la V de Aiken, con un resultado promedio de 0,95. Obtuvo un nivel de correlación moderado a través del coeficiente de correlación de Pearson y un alfa de Cronbach de 0,8. A través del análisis factorial exploratorio, se conformaron 3 dimensiones.

**Conclusiones:**

El instrumento se validó correctamente a través de 4 pruebas de validez. El nivel de conocimiento de los estudiantes de posgrado y docentes fue muy bueno en un 60,5%.

## Abstract (en)

**Objective:**

To build and validate an instrument to measure the level of knowledge about the Law that regulates the work of the dental surgeon in the Peruvian territory (Law No. 27878) of postgraduate students and teachers of the Stomatology Career of the Scientific University of Peru. South.

**Material and methods:**

: The sample consisted of 38 dental surgeons, participants in the postgraduate program (20) and professors (18) of the Stomatology Career at the Universidad Científica del Sur. A structured questionnaire of 15 questions was developed based on the articles of Law No. 27878.

**Results:**

It was approved by Expert Judgment and submitted to Aiken's V with an average result of 0.95. A moderate level of correlation was obtained through the Pearson Correlation Coefficient and a Cronbach's Alpha of 0.8. Through the Exploratory Factor Analysis, 3 dimensions were formed.

**Conclusions:**

The instrument was correctly validated through 4 validity tests. The level of knowledge of postgraduate students and teachers was very good in 60.5%.

## INTRODUCCIÓN

La odontología, como ciencia, ha evolucionado con el paso del tiempo. A través de los años, se han implementado nuevas tecnologías en el diagnóstico y tratamiento de los pacientes que acuden a los servicios de estomatología. Esta última es considerada una profesión médica desde la promulgación de la Ley N.° 16447, en 1967 ^1^, basada en una suma de conocimientos que permiten tomar decisiones sobre la salud oral de un individuo ^2^. La práctica estomatológica, también llamada acto odontológico ^(2, 3)^, no es otra cosa que la aplicación de habilidades y conocimientos, obtenidos de la evidencia científica, para realizar un servicio a favor de la salud bucodental de un paciente ^4^. El cirujano dentista se enfrenta a una realidad diferente a la de hace algunos años. La relación estomatólogo-paciente es fundamental en la toma de decisiones y en la cooperación del paciente para recibir un tratamiento ^5^; sin embargo, esta relación se ha deteriorado ^6^. Los factores más relevantes que han afectado dicha relación incluyen la incapacidad del profesional -cuyos actos transgredan la Lex Artis médica ^3^^,^^7^-, los errores de diagnóstico o planificación, las fallas en la comunicación entre el estomatólogo y el paciente, el no cumplir con sus expectativas, etc. ^8^.

Además, enfrenta una situación extra, la cual está basada en la propia naturaleza de su profesión. Se debe, principalmente, a que el profesional no trabaja solo, sino que requiere de un equipo que lo asista ^9^. Este equipo suele estar conformado por el cirujano dentista, los asistentes dentales, los recepcionistas y los colegas. En este ámbito laboral, el profesional debe entender que sus trabajadores y colegas también deben cumplir el marco legal, por lo que recae en él, como cabeza del equipo, asegurarse de su cumplimiento ^10^^,^^11^.

Esto ha dado como resultado un aumento del número de quejas y denuncias a cirujanos dentistas por parte de pacientes y empleados ^10^^,^^12^. En México, las demandas aumentaron un 22% entre 1996 y 2000 ^12^. En nuestro país, el número de cirujanos dentistas ha aumentado rápidamente y eso ha traído consigo un aumento en la trasgresión de la Lex Artis, así como en el número de denuncias realizadas por pacientes ^13^.

Toda profesión es regulada por leyes y normas éticas para que su ejercicio se realice de manera adecuada, y la odontología no es la excepción. A esta suma de normas aplicadas a una profesión se le conoce como deontología ^13^^-^^16^. La legislación de la profesión bucodental se realiza desde siglos atrás. En nuestro país, en 1997, se redactó la Ley N.° 26842, llamada Ley General de la Salud, la cual manifiesta que los profesionales de la salud son responsables de cualquier injuria que ocasionen en el paciente por un incorrecto ejercicio de su profesión ^3^^,^^4^^,^^17^. En 2002, se promulgó la Ley N.° 27878, llamada Ley de Trabajo del Cirujano Dentista, la cual contiene normas y estatutos que el profesional estomatológico debe seguir para el correcto ejercicio de su profesión ^18^. Dicha ley se ha visto modificada con el paso de los años a través de decretos supremos en los años 2005, 2016 y 2020 ^18^.

Es de vital importancia entender que la legislación peruana incluye las obligaciones que el odontólogo no puede pasar por alto y que se encuentran en el Código de Ética y Deontología del COP, en la Ley de Trabajo del Cirujano Dentista, en el Reglamento de Sanciones e Infracciones de SUSALUD en las Normas Técnicas de Salud N.° 113 modificada del Minsa, entre otras ^19^.

El papel que juega la legalidad en la práctica estomatológica es tal que no solo incluye el ejercicio ético, sino que facilita la determinación de los grados de responsabilidad civil, penal o administrativa ^2^, así como la disminución o erradicación del ejercicio ilegal de la profesión, lo cual no solo tiene impacto en el ejercicio per se, sino también en la perspectiva que tienen los pacientes sobre los odontólogos ^20^.

Ángeles ^3^ realizó un estudio para evaluar el nivel de conocimiento de los estudiantes de posgrado de la UNMSM sobre la normativa que regula la profesión del cirujano dentista en el Perú. El resultado fue que el mayor porcentaje de estudiantes tenía un nivel de conocimiento regular. Peña ^2^ realizó un estudio para evaluar el nivel de conocimiento de los estudiantes de posgrado de la Universidad Inca Garcilaso de la Vega y obtuvo como resultado que el 60% de los encuestados mostró un nivel de conocimiento regular. Pérez ^14^ llevó a cabo un estudio en Guatemala para medir el nivel de conocimiento de 96 cirujanos dentistas sobre sus derechos y obligación según la ley. Como resultado, se obtuvo que existe un bajo nivel de conocimiento sobre las leyes de Guatemala. Gambhir ^21^, en 2015, realizó un estudio en Punjab (India) para evaluar el nivel de conocimiento sobre el Acta de Protección al Consumidor en 280 cirujanos dentistas y halló que el 50% tuvo un bajo nivel de conocimiento.

Por tanto, el propósito de la presente investigación se traduce en la elaboración del primer cuestionario que evalúa el nivel de conocimiento de los cirujanos dentistas a nivel nacional (Perú) y que ha sido validado a través de los tres criterios establecidos.

## MATERIALES Y MÉTODOS

La población del estudio estuvo conformada por los docentes de pregrado y posgrado, y por estudiantes de posgrado de la Carrera de Estomatología de la Universidad Científica del Sur. La población del estudio se determinó mediante un recuento de todos los estudiantes de posgrado matriculados en el Ciclo Académico 2021-II, así como de los docentes de pre y posgrado que se encontraban trabajando en dicho ciclo.

La muestra se determinó mediante un muestreo no probabilístico. Se construyó un cuestionario de 15 preguntas de opción múltiple a partir de los artículos de la Ley N.° 27878, Ley de Trabajo del Cirujano Dentista, las cuales abordaron los temas más relevantes y que se prestaban a ser evaluados en el formato de opción múltiple. Contiene un área para los datos del estudiante o docente, y una para las preguntas. Cada pregunta respondida correctamente tiene la valoración de 1 punto, lo que origina la siguiente escala de evaluación, denominada Puntaje total: Muy malo: 0-3 puntos; Malo: 4-6 puntos; Regular: 7-9 puntos; Bueno: 10-12 puntos; Muy bueno: 13-15 puntos.

Para iniciar, se solicitó la autorización y permisos correspondientes al Departamento Académico de Salud y Vida, al posgrado de la carrera de Estomatología y al Comité de Ética de la Universidad Científica del Sur.

Las preguntas del cuestionario deben ser relevantes para el propósito evaluativo del estudio. Se elaboró la ficha de evaluación para el juicio de expertos, en la cual, para cada ítem, se utilizó una valoración de las afirmaciones en la escala de Likert de dos puntos para expresar mayor o menor acuerdo con los criterios del cuestionario.

Para evaluar la validez de contenido, se realizó un juicio de expertos. Se contó con un total de 5 expertos, a quienes se les envió el cuestionario y la ficha a través de su correo electrónico, para que evaluaran cada una de las preguntas. Los expertos colocaron en cada pregunta el puntaje de 0 o 1, dependiendo de si estaban de acuerdo o no con el ítem según sus criterios de valoración. Sus respuestas fueron brindadas en formato Word y se agruparon en una tabla de Excel.

Para evaluar las preguntas del cuestionario, se utilizó la V de Aiken. Para esto, se obtuvo el promedio simple del porcentaje de profesores que consideraron que la pregunta cumple con los criterios explicados. Se consideraron para la prueba solo preguntas con un índice de calidad igual o superior a 0,80. Estos datos fueron registrados en una matriz elaborada para la evaluación mediante la V de Aiken. Se realizó el análisis factorial exploratorio, el cual, usando un análisis de componentes principales, es una forma de resumir toda la data que se obtiene. Establece si existen preguntas que pueden ser agrupadas en dimensiones. Las dimensiones se ordenan según la varianza que pueden describir, de esta manera son útiles para disminuir la dimensionalidad (la cantidad de dimensiones en las que se puede agrupar) de la data.

Se basa en la reproducción de los resultados obtenidos en pruebas repetidas con la misma técnica. En esta etapa del proceso de validación, se aplicó el instrumento para evaluar a los estudiantes de posgrado. Los resultados fueron revisados a través de la prueba de alfa de Cronbach. Se considera confiable el cuestionario si se encuentra en un rango estadístico mayor o igual a 0,80.

El cuestionario fue enviado de manera virtual a través de Google Forms a los estudiantes de posgrado y a través del correo electrónico a los docentes. El consentimiento informado estuvo anexado al inicio del cuestionario y constó de 2 secciones. En la primera sección, se le brindó al participante toda la información del proyecto, así como los beneficios que puede obtener y la protección de sus datos personales. En la segunda sección, el participante pudo seleccionar entre dos alternativas si desea o no dar su consentimiento. De darlo, pudo continuar con las siguientes preguntas; de lo contrario, el cuestionario se cerró. 

Para demostrar la confiabilidad del cuestionario, se volvió a aplicar el instrumento a los estudiantes una semana después de haber aplicado el primero, a la espera de obtener los mismos resultados. Se seleccionó a un 50% de los estudiantes de forma voluntaria y se les aplicó el cuestionario por segunda vez, aunque de antemano se modificó el orden de las preguntas y las alternativas. Los datos fueron analizados mediante el coeficiente de correlación de Pearson. El coeficiente obtenido se categorizó según Hernández *et al*. ^22^, en su trabajo sobre el uso adecuado del coeficiente de correlación de Pearson.

La base de datos se registró en una hoja de cálculo del programa Excel. El análisis estadístico se realizó después de obtener el consenso final sobre las preguntas realizadas en el juicio de expertos. En este estudio se utilizó el programa estadístico R Studio. Con los resultados de la valoración de contenido por parte de los expertos, se utilizó el estadístico V de Aiken (rango aceptable de ≥ 0,80). Para determinar la validez del constructo, se procedió a la realización de un análisis factorial. Luego, se determinó la confiabilidad del instrumento mediante el cálculo del alfa de Cronbach (se consideró un rango aceptable ≥ 0,80) y, para la prueba test-re-test, se aplicó el coeficiente de correlación de Pearson.

Se solicitó la autorización de la Carrera de Estomatología de la Universidad Científica del Sur, así como del Departamento Académico de Salud y Vida. Se colocó toda la información del estudio antes que los participantes pudiesen escribir sus nombres y se guardó confidentemente la información de cada participante (nombre, correo electrónico, etc.). El consentimiento informado fue realizado a modo de pregunta antes de que pudiesen responder las demás preguntas.

## RESULTADOS

**Figura 1:**
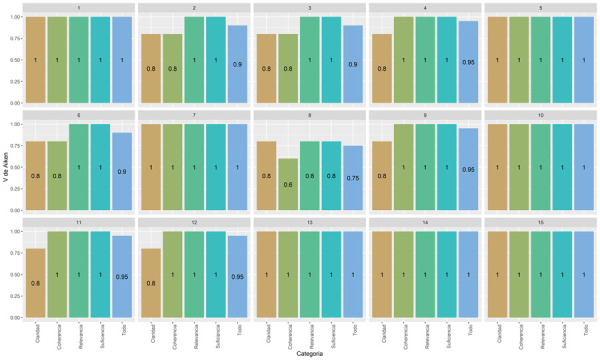
Evaluación de la validez por medio de la V de Aiken

Se envió el cuestionario con la ficha de evaluación vía *e-mail* a los 5 expertos y se recibió las 5 respuestas de la misma manera. Se elaboró una matriz en Excel con los puntajes de las cinco fichas y se llevó la data al *software* R Studio. Se utilizó la V de Aiken y se obtuvo como resultado valores que oscilaban entre 0,75 y 1 (Fig. 1), lo cual indica un nivel de validez aceptable. La moda de los datos fue 1 y el promedio, 0,95.

La muestra del estudio se determinó a través del recuento de los estudiantes y docentes, y tuvo un total de 42 participantes. Se utilizó un muestreo no probabilístico. A través del consentimiento informado (Tabla 1) y de los criterios de selección (Tabla 2), se obtuvo un total de 38 participantes. Se excluyó a 3 participantes que llevaron sus estudios de pregrado en una universidad extranjera. Si bien es cierto que, para trabajar dentro del territorio peruano deben conocer la normativa; no obstante, supone un diferencial con respecto a los que han llevado un curso sobre la ley de su país.

**Tabla 1:** Consentimiento de los participantes para ser parte de la investigación

Consentimiento	n	fi (%)
Dio consentimiento	41	97,6
No dio consentimiento	1	2,4
Total:	42	100

n: frecuencia fi: frecuencia relativa

**Tabla 2:** Estudios de pregrado de los participantes

Estudios de pregrado	n	fi (%)
Universidad peruana	38	92,7
Universidad extranjera	3	7,3
Total	41	100

n: frecuencia fi: frecuencia relativa

Para evaluar la confiabilidad, se procedió a realizar la prueba de alfa de Cronbach a partir de las preguntas y respuestas obtenidas. El resultado fue bueno, pues se obtuvo un alpha de 0,8, teniendo como límite superior 0,89 y como límite inferior, 0,72 (Tabla 3). 

**Tabla 3:** Evaluación de la confiabilidad con el test alfa de Cronbach

Alpha	95%-LB	95%-UB
0,8	0,72	0,89

**Figura 2:**
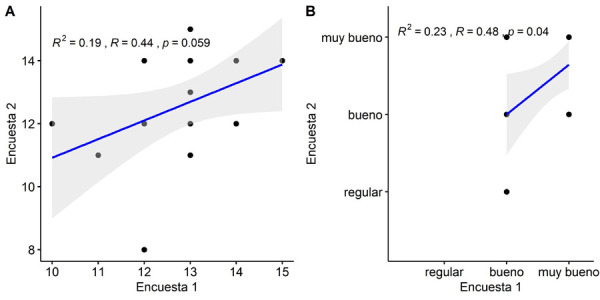
Correlación de Pearson. Izquierda: comparación de cuestionarios a nivel de puntaje. Derecha: comparación de cuestionarios a nivel de categoría.

Para evaluar la reproducibilidad del cuestionario, se procedió a enviarlo nuevamente al 50% de los encuestados, una semana luego de que respondieran el cuestionario anterior. Tras obtener las 19 respuestas, se procedió a realizar la prueba de correlación de Pearson. Se trabajó con un alfa de 0,05. Se obtuvo como resultado un valor R de 0,44, cuando se compararon los dos cuestionarios a nivel de puntaje, y un valor R de 0,48, a nivel de categoría, ambos resultados muy similares. Esto indicaría un nivel de correlación moderada (Figura 2).

**Figura 3:**
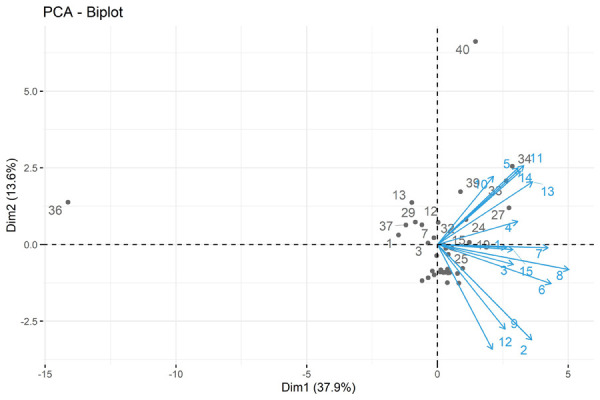
PCA (análisis de componentes principales)

Para evaluar la validez del constructo, se realizó el análisis factorial exploratorio, a través del análisis de componentes principales, para conformar y analizar las dimensiones del cuestionario. Se obtuvo como resultado la conformación de dos principales dimensiones (Figura 3). 

**Figura 4:**
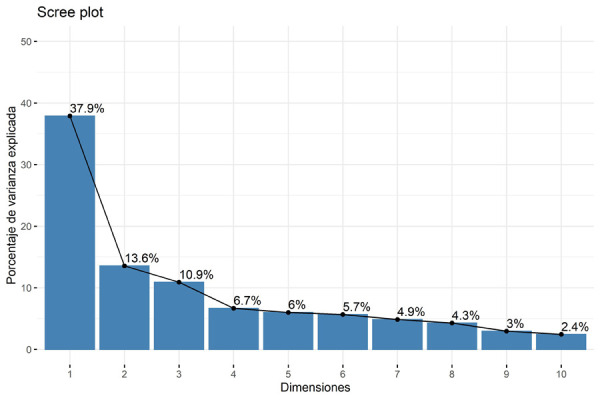
Dimensiones a ser formadas según el porcentaje de varianza a ser explicada. Las dimensiones 1 y 2 son las que explican el mayor porcentaje.

Las dimensiones se ordenan según la varianza que pueden describir, por lo que es útil para disminuir la dimensionalidad (la cantidad de dimensiones en las que se puede agrupar) de la data (Figura 4).

**Figura 5:**
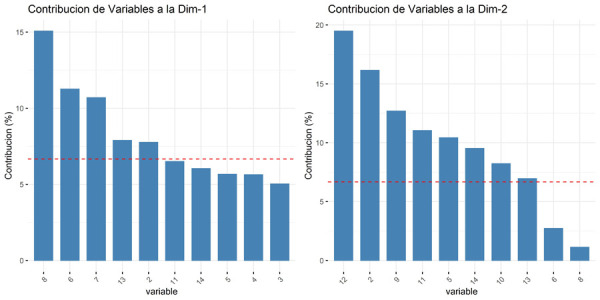
Contribución de los ítems a las principales dimensiones del cuestionario

Se estableció el orden de las preguntas según su contribución individual con cada dimensión (Figura 5). En la primera dimensión, se agruparon los ítems 8, 6, 7 y 13, los cuales abarcan los temas de requisitos para el ejercicio de la profesión y el trabajo administrativo. Por lo tanto, fue denominada Requisitos para el ejercicio legal de la profesión y ámbito administrativo. La segunda dimensión abarcó los ítems 12, 2, 9, 11, 5 y 14, que incluyen temas sobre el trabajo estomatológico, las especialidades reconocidas y las modalidades laborales; por lo tanto, fue llamada Acto estomatológico, especialización y modalidades laborales. Los ítems restantes (1, 3, 4, 10 y 15) corresponden a temas intrínsecos del cirujano dentista, tales como las actividades, funciones y derechos del odontólogo, así como las horas de la jornada ordinaria laboral. Se agruparon en una tercera dimensión denominada Cirujano dentista y su jornada ordinaria. 

Finalmente, se evaluó el resultado de los 38 participantes, se organizaron las respuestas en Excel y se realizó el análisis correspondiente a través de tablas de frecuencia. La edad de los participantes fue agrupada en intervalos, siendo el intervalo más frecuente el de 31 a 40 años, con un 34,2% (Tabla 4). 

**Tabla 4:** Intervalo de edades de los participantes

Intervalo de edades	n	%
20-30 años	11	28,9
31-40 años	13	34,2
41-50 años	11	28,9
51-60 años	3	7,9
Total	38	100

n: frecuencia fi: frecuencia relativa

El género con mayor frecuencia fue el femenino con un 68,4%, mientras que el masculino solo presentó un 31,6% (Tabla 5).

**Tabla 5:** Género de los participantes

Género	n	%
Femenino	26	68,4
Masculino	12	31,6
Total	38	100

n: frecuencia fi: frecuencia relativa

En cuanto a la cantidad de docentes y estudiantes, fueron evaluados 18 docentes (47,4%) y 20 estudiantes de posgrado (52,6%) (Tabla 6).

**Tabla 6:** Frecuencia de los estudiantes y docentes

Participante	n	%
Estudiante de posgrado	20	52,6
Docente	18	47,4
Total	38	100

n: frecuencia fi: frecuencia relativa

El nivel de conocimiento obtenido fue, en su mayor parte, Muy bueno, con un 60,5%, seguido por un nivel Bueno (34,2%). Los niveles regular y malo solo obtuvieron un resultado cada uno (Tabla 7).

**Tabla 7:** Nivel de conocimiento de los participantes

Nivel de conocimiento	n	%
Muy malo	0	0
Malo	1	2,6
Regular	1	2,6
Bueno	13	34,2
Muy bueno	23	60,5
Total	38	100

n: frecuencia fi: frecuencia relativa

Al dividir a los participantes entre estudiantes de posgrado y docentes de la Carrera de Estomatología de la Universidad Científica del Sur (Tabla 8), un 36,8% ^14^ corresponde a los docentes que obtuvieron un puntaje Muy bueno; mientras que un 23,7% ^23^ correspondió a los estudiantes de posgrado que obtuvieron el puntaje de Muy bueno. Ambos grupos conforman el 60,5% mencionado en la tabla anterior.

**Tabla 8:** Nivel de conocimiento de los docentes y estudiantes de posgrado

	Docentes		Posgrado		TOTAL	
Nivel de conocimiento	n	fi (%)	n	fi (%)	n	fi (%)
Muy malo	0	0	0	0	0	0
Malo	0	0	1	2.6	1	2.6
Regular	0	0	1	2.6	1	2.6
Bueno	4	10.5	9	23.7	13	34.2
Muy bueno	14	36.8	9	23.7	23	60.5
Total	18	47.3	20	52.6	38	100

## DISCUSIÓN

En este estudio, se estableció un instrumento que demostró su validez a través de cuatro pruebas, siendo el primer estudio del país en validar su instrumento a través de su contenido, confiabilidad y constructo. La literatura previa registrada en el país documenta un número menor de pruebas de validez. Tal es el caso de Monge ^15^ y Ángeles ^3^, los cuales validaron sus respectivos cuestionarios mediante dos pruebas de validación (juicio de expertos y alfa de Cronbach). Vásquez ^13^ utilizó también tres pruebas de validación, dos de ellas de validación de contenido (juicio de expertos y coeficiente de validación de contenido a través del modelo de Lawshe) y una de confiabilidad (alfa de Cronbach). Peña ^2^ y Reyes ^19^ utilizaron solo una prueba de validación en sus respectivas investigaciones (juicio de expertos). Pérez ^14^ no documentó ninguna prueba de validez efectuada sobre su instrumento.

La valoración por juicio de expertos no brinda un valor que pueda ser cuantificado directamente, sino que, a través de este análisis, se puede realizar una prueba que cuantifique el resultado, tal y como lo hacen los coeficientes de validación de contenido ^23^. La validez de contenido fue evaluada a partir de la V de Aiken, la cual cuantificó cada pregunta según la valoración de los jueces. Es un coeficiente que permite cuantificar la relevancia de los ítems respecto a un dominio de contenido a partir de las valoraciones de N jueces. Los estudios previos realizaron el juicio de expertos, mas no documentaron el uso de algún análisis cuantificador. Esos fueron los casos de Monge ^15^, Ángeles ^3^, Peña ^2^ y Reyes ^19^. Vásquez ^13^, además de utilizar el juicio de expertos, usó el modelo de Lawshe para cuantificar el resultado obtenido. Tal modelo fue propuesto para indicar un índice para la validez de contenido; no obstante, una gran desventaja de este índice es que precisa un gran número de jueces con un nivel de acuerdo casi unánime ^24^.

La validez de constructo es un paso que fue omitido en la mayor parte de las investigaciones en las que se diseñó el instrumento. Las tres dimensiones conformadas a través el análisis factorial exploratorio explicaban de mejor manera la varianza y agruparon de manera correcta los ítems. La formación de las dimensiones de los instrumentos de las literaturas previas no fue documentada; por lo tanto, podría interpretarse como una formación a criterio del autor. 

La confiabilidad fue evaluada, tanto en esta investigación como en la literatura previa, mediante el alfa de Cronbach. Monge ^15^, Ángeles ^3^ y Vásquez ^13^ obtuvieron un valor de 0,852, 0,808 y 0,812 en sus respectivos estudios. Estos resultados son similares a los obtenidos en esta investigación. La prueba test-re-test no fue evaluada en la literatura previa. La correlación de ambos cuestionarios fue moderada; no obstante, cabe resaltar que, si se realizase tal análisis con muestras de mayor tamaño, los resultados serían mucho más favorables.

Tras ser evaluado, el cuestionario arrojó como resultado general que el nivel de conocimiento de los estudiantes de posgrado y los docentes es Muy bueno en un 60,5%, seguido por un nivel Bueno, con un 34,2%, lo cual muestra una gran diferencia con respecto a investigaciones previas. Monge ^15^ obtuvo un resultado regular, con un 71%; Ángeles ^3^ obtuvo un 48.9% regular; Peña obtuvo un resultado regular, con un 60%; Reyes ^19^ y Vásquez ^13^ obtuvieron puntajes del 70% y el 71%, respectivamente. En Guatemala, Pérez ^14^ obtuvo un 64% malo. 

Esto puede ser explicado principalmente por dos factores que conformarían las principales limitaciones de este estudio. El primero consiste en la muestra, cuyo tamaño fue menor en comparación con la literatura previa. El segundo factor fue el mismo formato del cuestionario. En la literatura previa, los cuestionarios fueron realizados de manera presencial, acudiendo al centro de trabajo de los cirujanos dentistas o a la clínica odontológica de la universidad en la que realizaron su investigación. El cuestionario en esta investigación se realizó de manera virtual, debido a la pandemia de COVID-19. Esto supone una clara diferencia debido a un componente psicológico, puesto que es muy distinto realizar un cuestionario desde la comodidad de un hogar, en el horario a preferencia del participante, sin límite de tiempo, que realizarlo con un tiempo límite y en un momento determinado del día.

La principal limitación del estudio consiste en la población. Debido a la pandemia de COVID-19 las clases se dictaron de manera virtual y, por ende, hubo un descenso en la cantidad de estudiantes de posgrado en todas las especialidades. Como consecuencia, se obtuvo una población bastante reducida. Otro posible sesgo fue la respuesta aleatoria de las preguntas o la búsqueda de las respuestas de manera virtual, ante el desconocimiento de ellas, por lo que se les instó a responder de manera completa el cuestionario y que lo realicen a conciencia.

## CONCLUSIONES

El cuestionario diseñado en esta investigación fue validado correctamente para medir el nivel de conocimiento de los estudiantes de posgrado y docentes de la Universidad Científica del Sur sobre la Ley de Trabajo del Cirujano Dentista. La validez del contenido del instrumento fue buena. La confiablidad del instrumento fue buena. La prueba de validez de constructo del instrumento permitió conformar 3 dimensiones, otorgándole estructura lógica al cuestionario. El nivel de conocimiento de los estudiantes de posgrado y docentes de la Universidad Científica del Sur sobre la Ley de Trabajo del Cirujano Dentista fue muy bueno en un 60,5%.

## Fuente de financiamiento:

Autofinanciado
